# The Roles of TOPK in Tumorigenesis and Development: Structure, Mechanisms, Pathways, and Therapeutic Implications

**DOI:** 10.7150/ijbs.122960

**Published:** 2026-01-01

**Authors:** Mengyu Zhao, Min Zhang, Mengrui Liang, Xinru Wang, Qipan Feng, Shengjie Wu, Xueli Tian, Ding Ding, Xiang Li, Kangdong Liu, Mee-Hyun Lee, Ran Zhao, Zigang Dong

**Affiliations:** 1 Department of Pathophysiology, School of Basic Medical Sciences, Zhengzhou University, Zhengzhou 450001, China.; 2 China-US (Henan) Hormel Cancer Institute, Zhengzhou 450008, China.; 3 Tianjian Laboratory of Advanced Biomedical Sciences, Zhengzhou 450052, China.; 4 Department of Clinical Research and Translational Medicine, The Third Affiliated Hospital of Zhengzhou University, Zhengzhou,450002, China.; 5 College of Korean Medicine, Dongshin University, Naju 58245, Republic of Korea.

## Abstract

TOPK (T-LAK cell-originated protein kinase), also known as PDZ-binding kinase, is a serine/threonine kinase belonging to the mitogen-activated protein kinase family. It is a critical regulator of essential cellular processes, including survival, proliferation, apoptosis, inflammation, and autophagy. As an oncogenic kinase, TOPK is predominantly expressed in actively proliferating cells, where its dysregulation contributes to the pathogenesis of various cancers. Through phosphorylation, TOPK activates key signaling pathways such as ERK/RSK/c-Jun, which in turn promote cancer cell proliferation, migration, and resistance to apoptosis. Furthermore, TOPK has been implicated in the regulation of the tumor microenvironment and immune evasion. This review provides an in-depth examination of the molecular structure of TOPK, the role of TOPK in tumorigenesis, and the underlying mechanisms that support its oncogenic activity. Given its central role in cancer progression, TOPK represents a promising candidate for novel cancer therapies. Additionally, we explore the therapeutic potential of targeting TOPK in cancer treatment, highlighting ongoing research efforts and the challenges in translating TOPK inhibition into clinical practice.

## Introduction

PDZ-binding kinase/T-LAK cell-derived protein kinase (PBK/TOPK) encodes a serine/threonine protein kinase that plays as a pivotal role in regulating key cellular processes, including survival, proliferation, growth, apoptosis, and inflammation 1, 2. Under physiological conditions, TOPK expression is primarily restricted to tissues with high proliferative activity. It is abundantly expressed in testis, particularly in spermatogenic germ cells, and in a few normal tissues such as the placenta, and T-LAK cells 3, 4. In contrast, its expression is minimal or nearly absent in non-proliferative normal tissues such as the adult brain, except for regions containing rapidly dividing progenitor cells, including the subependymal zone and the outer layer of the early postnatal cerebellum 5. In pathological contexts, however, TOPK is markedly upregulated in malignant tumor cells. Elevated TOPK expression has been observed across various cancer types, and is often associated with tumor aggressiveness and poor clinical prognosis 4. As a member of the mitogen-activated protein kinase kinase (MAPKK) family, TOPK exerts its biological functions by phosphorylating downstream targets through its kinase domain, thereby modulating signaling pathways relate to proliferation, apoptosis, inflammation, and autophagy. Given its dual presence in proliferative normal tissues and tumors, TOPK has emerged as a promising biomarker and therapeutic target in oncology. It contributes to tumor progression through multiple mechanisms, including the regulation of signal transduction, cellular metabolism, and immune modulation. Targeted TOPK has shown potential in inhibiting cancer growth, making it an attractive target. Consequently, understanding the molecular mechanisms underlying TOPK activation and regulation is of great significance for elucidating its roles in cancer biology. In this review, we comprehensively discuss the structure, expression patterns, biological functions, molecular mechanism, and inhibitors of TOPK in cancer. A deeper understanding of its multifaceted regulatory roles will not only advance our knowledge of cellular signaling and disease pathogenesis but also pave the way for novel precision medicine approaches and targeted cancer therapies.

## The structure of TOPK

**Figure 1:**
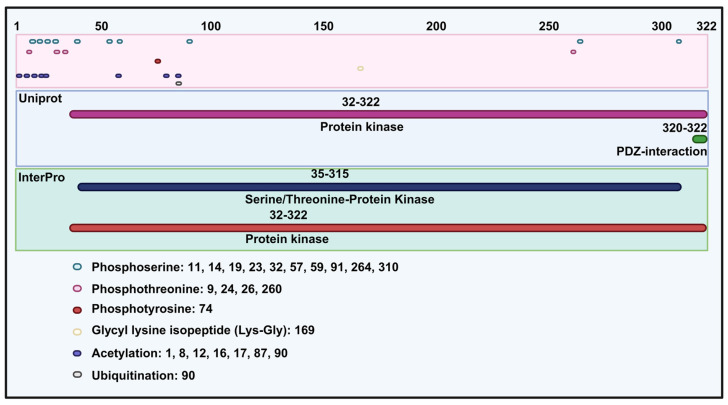
Protein structure of TOPK and phosphorylation, glycyl lysine isopeptide, acetylation and ubiquitination site of TOPK.

TOPK, a member of MAPKK, shares structural similarities with other kinases in this family, particularly in its serine/threonine kinase subdomains, which are essential for its enzymatic activity. It contains 322 amino acids and has a molecular weight of 36.09 kDa. Specifically, according to the UniProt database (https://www.uniprot.org/) and InterPro database (https://www.ebi.ac.uk/interpro/), TOPK includes a protein kinase domain (32-320 amino acids) and PDZ-interaction region (Figure 1). Structurally, TOPK consists of 11 alpha helices and 11 beta sheets. In addition, molecular modeling reveals that the N-terminal domain is more hydrophobicity than the C-terminal domain, with nonpolar amino acids enriched in the C-domain 6. With respect to post-translational modification, TOPK is mainly regulated through phosphorylation of serine, threonine, and N6-acetyllysine (Figure 1). The N-terminal region of TOPK contains a conserved phosphorylation site at threonine 9 (Thr9), which is phosphorylated by cdc2/cyclin B during mitosis 7. Functionally, phosphorylation at Thr9 contributes to cytokinesis, chromosomal segregation, and the regulation of the DNA damage response 3, 8, 9. Inhibition the kinase activity of TOPK at this site has been shown to suppress tumor growth 10, 11. Moreover, another phosphorylation site is located at threonine 198 (Thr198), phosphorylated by an unidentified MAPKKK. This site, residing in the ATP-binding pocket, is crucial for the catalytic activity of TOPK and remains phosphorylated throughout the cell cycle 3, 12. TOPK also contains a C-terminal PDZ-binding motif (T/SXV), enabling interactions with PDZ domain-containing proteins such as hDlg 13, and with proteins like PRC1 to facilitate spindle formation and cytokinesis 14.

Regarding conformational changes, different pH conditions induce a transition of PBK between dimers and monomers. Under alkaline conditions, it forms an inactive dimer, with the C-terminal region being structurally conserved and the N-terminal region showing significant variability 15. Notably, the only available crystal structure of TOPK is a double mutant (T9E and T198E) dimer with a distorted N-leaf conformation 16. Furthermore, Markov state model (MSM) analysis suggests that wild-type TOPK can partially open even without phosphorylation, while the T9E and Y74E mutations, which accelerate the opening transition, enhance global activity. Based on these findings, two potential phosphorylation regulation pathways are proposed: one where phosphorylation accelerates the open transition of the closed conformation, and another where phosphorylation inhibits the closed-to-open transition 17. Structural studies using templates such as 2F4J from the Protein Data Bank have provided insights into the 3D conformation of TOPK, its binding sites, and interactions with inhibitors such as HI-TOPK-032 10, 18. Overall, the structural features of TOPK highlight its crucial role as a mitotic kinase and its potential as a therapeutic target in cancer treatment, owing to its involvement in oncogenic signaling and tumor progression.

## Expression of TOPK in cancer

TOPK, an oncogenic protein, plays a critical role in regulating cell survival, proliferation, growth, apoptosis, and inflammation. Accumulating evidence indicates that TOPK is dysregulated in various cancers, and its expression levels correlate with poor prognosis and tumor aggressiveness. For instance, it is significantly upregulated in several tumor types, including adrenocortical carcinoma (ACC), bladder urothelial carcinoma (BLCA), breast invasive carcinoma (BRCA), cervical squamous cell carcinoma and endocervical adenocarcinoma (CESC), colon adenocarcinoma (COAD), lymphoid neoplasm diffuse large B-cell lymphoma (DLBC), esophageal carcinoma (ESCA), glioblastoma multiforme (GBM), brain lower grade glioma (LGG), liver hepatocellular carcinoma (LIHC), lung adenocarcinoma (LUAD), lung squamous cell carcinoma (LUSC), ovarian serous cystadenocarcinoma (OV), pancreatic adenocarcinoma (PAAD), rectum adenocarcinoma (READ), skin cutaneous melanoma (SKCM), stomach adenocarcinoma (STAD), thymoma (TYHM), uterine corpus endometrial carcinoma (UCEC), uterine carcinosarcoma (UCS). By contrast, it is downregulated in acute myeloid leukemia (LAML) and testicular cancer (TGCT) according to the GEPIA 2 database (http://gepia2021.cancer-pku.cn/) (Supplement Figure 2A) 19. Consistently, data from the TIMER 2.0 database (https://compbio.cn/timer2/) show that TOPK/PBK is upregulated in multiple cancers, including BLCA, BRCA, CESC, cholangio carcinoma (CHOL), COAD, ESCA, GBM, head and neck squamous cell carcinoma (HNSC), kidney chromophobe (KICH), kidney renal clear cell carcinoma (KIRC), kidney renal papillary cell carcinoma (KIRP), LIHC, LUAD, LUSC, PAAD, pheochromocytoma and paraganglioma (PCPG), prostate adenocarcinoma (PRAD), SKCM with metastasis, STAD, thyroid carcinoma (THCA), while showing reduced expression in LAML and TGCT (Supplement Figure 2B) 20-22. Furthermore, our summary of experimental data confirms that TOPK is notably expressed in bladder cancer, breast cancer, cervical cancer, cholangiocarcinoma, chordoma, colorectal cancer, esophageal squamous cell carcinoma (ESCC), gastric cancer, lung adenocarcinoma, lymphoma, oral cancer, osteosarcoma, and ovarian cancer, whereas it is underexpressed in clear hepatocellular carcinoma (Table 1). Notably, according to the research and Kaplan-Meier Plotter database (https://kmplot.com/) show that elevated expression of TOPK has been linked to increased tumor aggressiveness and poor prognosis across multiple cancers (Supplement Figure 2C) 23-27. In contrast, TOPK expression is reduced in TGCT, consistent with database analyses 28. In addition to expression levels, analysis of TOPK mutations shows significant correlation with cancer subtypes, such as Basal and LumA of BRCA, COAD, GBM, LIHC, LUAD, READ, and SKCM of primary tumors, according to the TIMER 2.0 database. Moreover, data from cBioPortal database (https://www.cbioportal.org/) reveals that TOPK mutations, included missense, fusion, and insertions, are present in cancers such as renal non-clear cell carcinoma, ovarian epithelial tumor, and glioblastoma (Supplement Figure 1A-B) 29-31. In conclusion, these findings further highlighting the key role of TOPK in cancer development, both through deregulated expression and genetic alterations (Supplement Figure 1-2).

## TOPK as a tumor promoter in cancer

**Figure 2:**
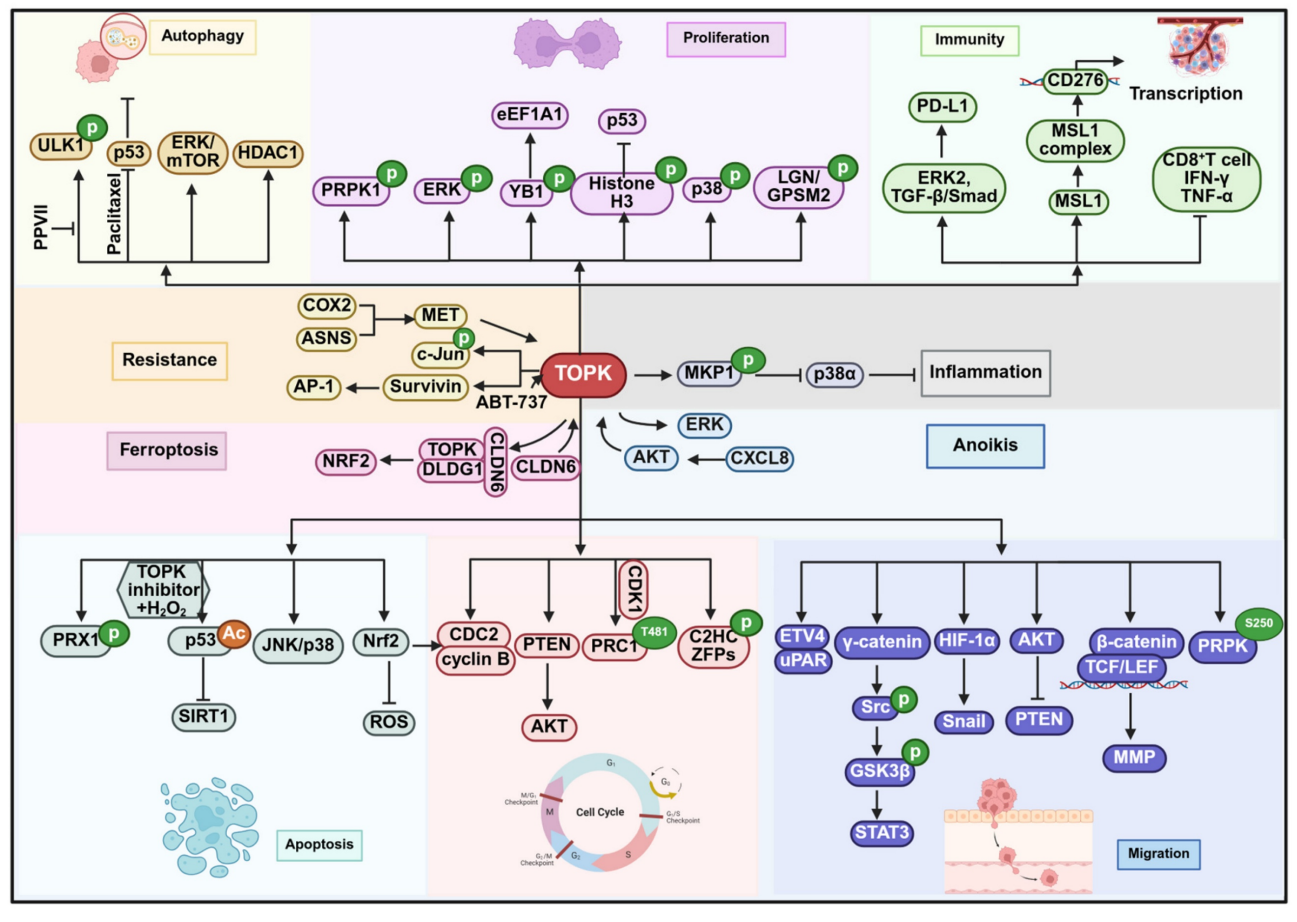
Functional roles and molecular mechanisms of TOPK in cancer development and progression.

**Figure 3:**
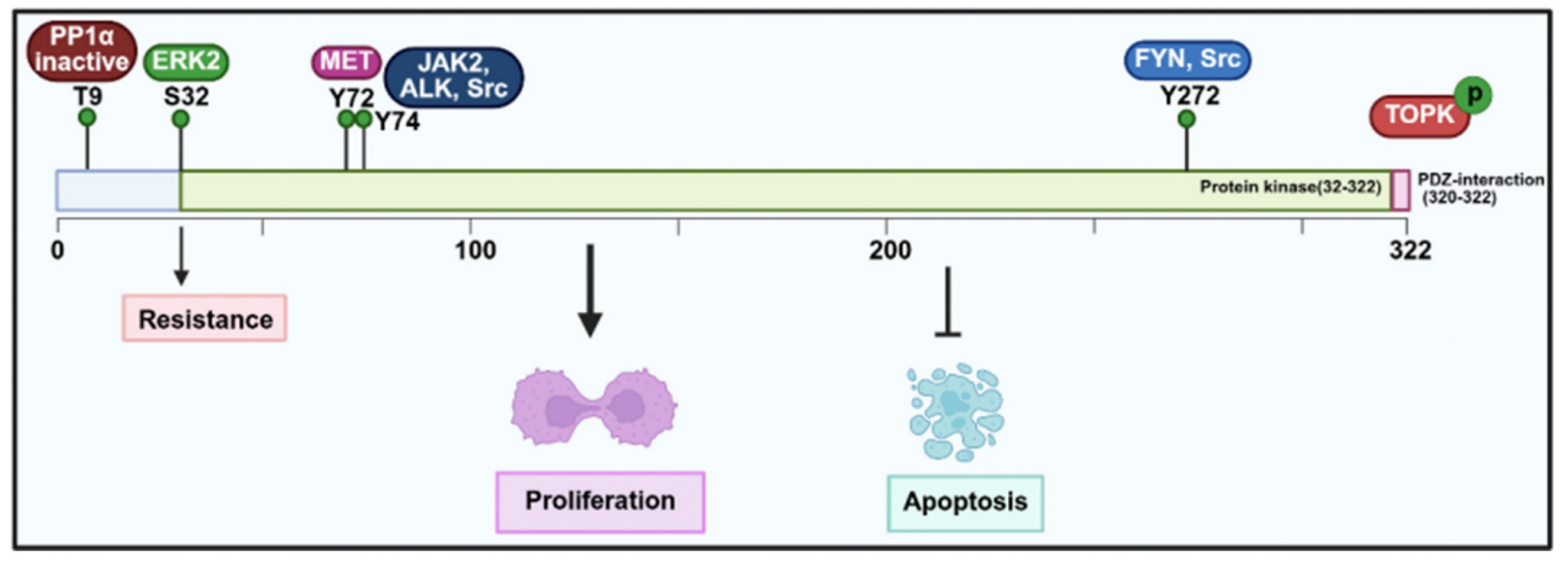
Regulatory mechanisms and biological functions of TOPK involved in tumorigenesis.

**Figure 4:**
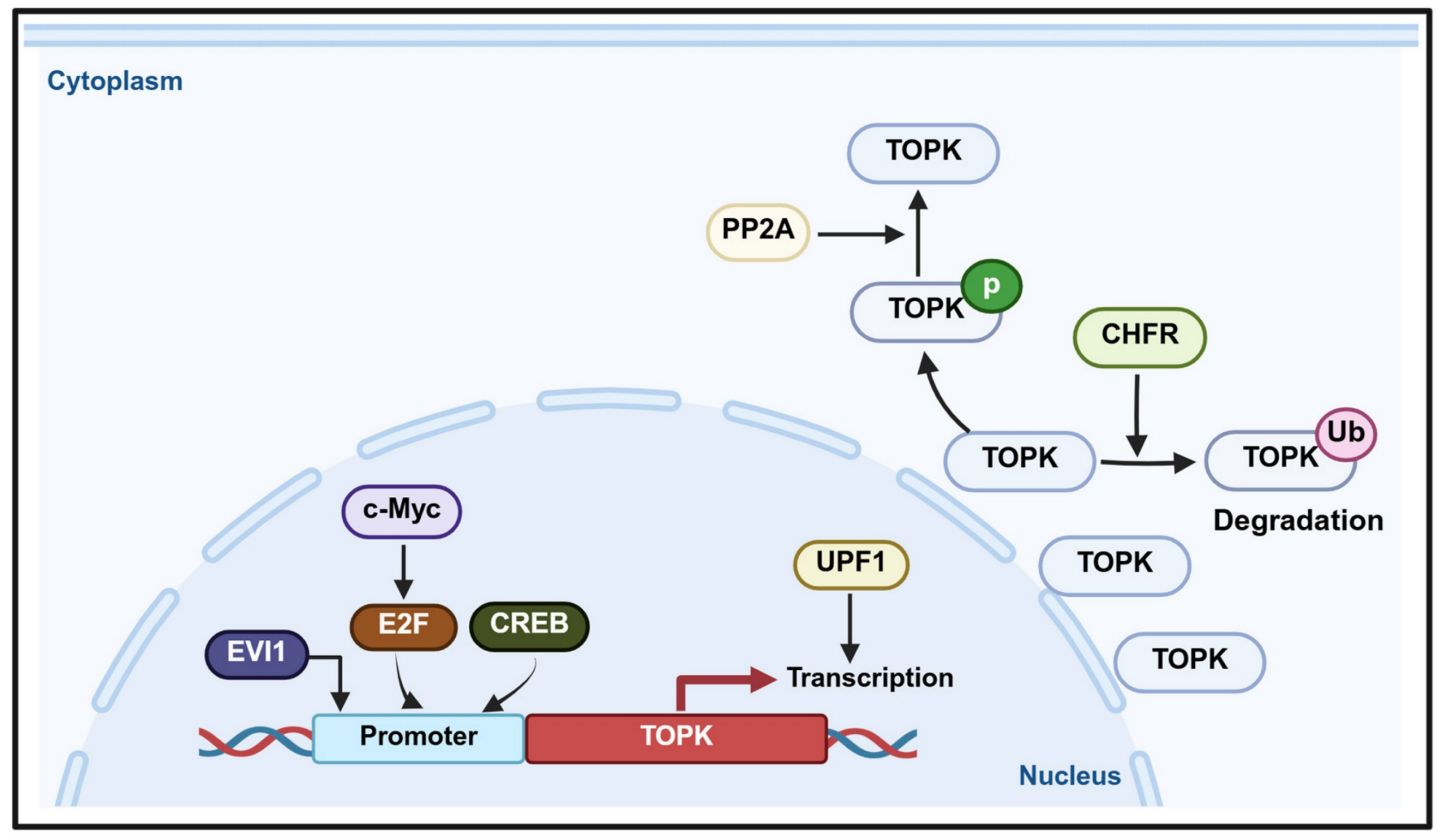
Regulation of the TOPK promoter and transcriptional activity.

As a mitotic kinase, TOPK regulates the timely progression of cells through the metaphase checkpoint during mitosis, ensuring proper cell cycle progression. Notably, the upregulation of TOPK in cancer is closely associated with tumor progression, including invasiveness, metastasis, and prognosis 23, 50. It also plays an integral role in chromosome segregation and cytokinesis by phosphorylating multiple targets (Figure 2-4) 51. In this section, therefore, we explore the expression patterns of TOPK in different cancers, its molecular functions in regulating cell cycle progression, apoptosis, and epithelial-mesenchymal transition (EMT), and its pivotal role in promoting tumor growth and metastasis. Moreover, we discuss the underlying mechanisms which TOPK contributes to cancer progression, such as its interactions with critical signaling pathways and involvement in DNA damage repair, autophagy, and immune responses.

### Role of TOPK in cell proliferation and tumor growth

Elevated TOPK expression has been linked to poor outcomes in various cancers (Table 1). For example, in breast cancer, high TOPK expression correlates with adverse clinicopathological features and poor prognosis 33, 34. Similarly, increased TOPK expression in oral cancer serves as a reliable prognostic marker for patient survival 47. Consistent with these findings, elevated TOPK expression has also been significantly associated with survival rates in chordoma and lymphoma 37, 44, 45. In addition, in renal cell carcinoma (RCC), particularly in advanced stages, TOPK overexpression is an independent prognostic factor, making it a potential marker for predicting patient outcomes 52. Interestingly, evidence indicates that TOPK is elevated in hematologic malignancies but significantly suppressed during TPA-induced differentiation of HL-60 cells, with decreased c-Myc phosphorylation 53. Functionally, increased expression of TOPK has been shown to enhance cell proliferation *in vitro* and drive tumor formation *in vivo* 54. Mechanistically, TOPK plays a key role in mitotic spindle assembly and function, particularly through phosphorylation at Thr9 by the Cyclin-Dependent Kinase 1 (CDK1)/cyclin B complex (Figure 2). This phosphorylation facilitates the recruitment of CDK1/cyclin B to the mitotic spindle, ensuring proper spindle dynamics during mitosis. The phosphorylation of TOPK is further regulated by CDK1, which inactivates protein phosphatase 1α (PP1α), thereby enabling TOPK activation (Figure 3) 51, 55. Moreover, TOPK interacts with microtubule-associated proteins (MAPs) to ensure accurate localization of CDK1/cyclin B to the spindle 7. Beyond this, PBK directly binds to cyclin-dependent kinase 5 (CDK5), and phosphorylation at Thr9 promotes EMT progression and proliferation of prolactinomas, thereby enhancing the stability of PBK. Moreover, phosphorylated p38 levels decrease with increasing Thr9 phosphorylation of TOPK both *in vivo* and *in vitro* 56. Downstream, TOPK activates signaling pathways such as MAPKs and ribosomal S-6 kinase (RSK), which drives cancer cell proliferation and metastasis. It also interacts with transcription factors including AP-1 and NF-κB, linking its kinase activity to cancer progression 57. In addition, the interaction between TOPK and upstream kinases such as Src increases its stability and activity through phosphorylation at Y74 and Y272, thereby promoting oncogenesis 58. PBK also upregulates c-Myc expression through phosphorylation of ERK1/2, whereas inhibitors like OTS514 block ERK1/2 phosphorylation and c-Myc transcriptional activity, thereby reversing PBK-induced proliferation and metastasis 59. Furthermore, the phosphorylation of TOPK by Janus kinase 2 (JAK2) at Tyr74 enhances its activity, driving tumor proliferation *in vivo* and carcinogenesis *in vitro* 60. TOPK also regulates the expression of Y-box binding protein 1 (YB1), a key oncoprotein involved in tumorigenesis. By phosphorylating YB1 at Thr89 and Ser209, TOPK enhances YB1-mediated transcription of eEF1A1, triggering the AKT/mTOR signaling pathway and promoting tumor growth 61. Similarly, TOPK and PTEN play critical roles in CHFR-mediated mitotic regulation, where TOPK inactivates PTEN and activates the AKT pathway, thus ensuring proper G2/M progression 62. Moreover, TOPK regulates H₂O₂-mediated signal transduction via its interaction with Prx1 and phosphorylation at Ser32, thereby modulating oxidative stress responses 63. Importantly, TOPK interacts with the DNA-binding domain of p53, suppressing its transcriptional activity and impairing tumor suppressor functions. In gastrointestinal cancers, TOPK overexpression is linked to cell proliferation and TP53 mutations, further emphasizing its role in oncogenesis 42, 64. TOPK also stabilizes Nrf2, a key regulator of oxidative stress, thereby promoting cell cycle progression and survival under stress conditions, such as in immature granulocytes 50. Moreover, TOPK enhances UVB-induced JNK1 activity, which is crucial for cell transformation mediated by H-Ras 65. LGN/GPSM2 plays a key role in cell division in breast cancer. PBK/TOPK targets Thr450 of LGN/GPSM2 during mitosis, suggesting that the PBK/TOPK-LGN/GPSM2 pathway could be a potential target for breast cancer therapies 66. PBK further enhances cell proliferation by phosphorylating histone H3 and inhibits colorectal cancer migration and invasion through CDH1 stabilization 67. Additionally, TOPK/PBK regulates C2H2 zinc finger proteins (ZFPs) during mitosis by phosphorylating C2H2 adaptor sequences *in vitro* 68.

By contrast, inhibiting TOPK in cancer cell lines significantly suppresses tumor growth *in vivo* 69. In HCC cells, TOPK inhibition results in cell cycle arrest and reduced colony formation, underscoring its role in cell proliferation and survival 70. Furthermore, oxidative stress induced by HI-TOPK-032 promotes cell apoptosis by activating the MAPK signaling pathway, highlighting its role of TOPK in regulating NPC growth 71. Inhibition of TOPK has been shown to suppress tumor growth and enhance treatment sensitivity 72, 73. Notably, knockdown of TOPK enhances radiosensitivity by altering cell cycle dynamics and increasing radiation-induced damage. Specifically, TOPK depletion disrupts the G1/S transition and G2/M arrest, leading to chromosomal aberrations, multinucleation, and apoptotic cell death 74. TOPK and maternal embryonic leucine zipper kinase (MELK) together regulate cancer cell proliferation and stem-like properties. Inhibiting either kinase reduces FOXM1 activity, suggesting that TOPK and MELK are promising therapeutic targets for renal cancer. Moreover, combined inhibition with OTS514 (TOPK inhibitor) and OTS167 (MELK inhibitor) enhances antitumor efficacy and may reduce side effects 72.

### Role of TOPK in tumor invasion and metastasis

Epithelial-mesenchymal transition (EMT) is a critical process that promotes cancer metastasis by enhancing cell mobility, invasiveness, and resistance to apoptosis 75. In this context, TOPK plays a central role in promoting EMT, thereby driving tumor invasion and metastasis. Specifically, TOPK facilitates EMT and tumor invasiveness by modulating multiple signaling pathways 76. Mechanistically, as a mitotic kinase, TOPK amplifies CDK1/cyclin B1-dependent phosphorylation of PRC1 at Thr481, which is essential for efficient cell division 14. It further regulates EMT in breast cancer through the TGF-β/Smad and NF-κB/Snail signaling pathways 76-79. In gastric cancer, FYN, a Src family kinase, interacts with TOPK to promote proliferation and metastasis by phosphorylating TOPK at the Y272 and HSPB1 80, 81. Moreover, TOPK promotes AKT phosphorylation while reducing PTEN levels, thereby enhancing cell migration and tumor progression through the PI3K/PTEN/AKT pathway 82. TOPK facilitates the metastasis of ESCC by activating the Src/GSK3β/STAT3 and ERK signaling pathway through γ-catenin 83. Under hypoxia conditions, TOPK upregulates HIF-1α expression, which promotes EMT and enhances the invasive potential of NSCLC cells 84. Similarly, in prostate cancer, TOPK activates the β-catenin-TCF/LEF pathway, leading to upregulation of MMP-2 and MMP-9, both of which are associated with tumor invasion and metastasis 77, 85. At the molecular regulation level, SRSF7 has been shown to enhance GBM cell proliferation and migration partly through m^6^A modification on PBK mRNA, with two m^6^A sites on PBK mRNA being directly regulated by SRSF7 86. In addition, TOPK interacts with PRPK to regulate colorectal cancer metastasis 87, while PBK overexpression promotes HCC metastasis via the ETV4-uPAR pathway 88. Finally, TOPK also enhances renal fibrosis through the SGK3/TOPK signaling axis, further driving EMT 89.

### Role of TOPK in apoptosis and autophagy

TOPK also plays a crucial role in apoptosis through its interactions with various molecules. For instance, TOPK interacts with histone H2AX, suppressing its phosphorylation in response to arsenic (As³⁺) and thereby promoting apoptosis in cancer cells. This strategy shows potential for melanoma therapy 9. Moreover, ALK phosphorylates TOPK at the Y74 site to promote cancer cell survival, while dual inhibition of ALK and TOPK significantly increases apoptosis in ALK-positive cancer cells both *in vitro* and *in vivo* 90. Similarly, inhibition of TOPK enhances hydrogen peroxide-induced apoptosis in ovarian granulosa cells by regulating the p53/SIRT1 axis, thereby promoting pro-apoptotic gene expression and cell death 91, 92. In chordoma, elevated TOPK expression reduced anti-apoptotic proteins such as Mcl-1 and Survivin, while increasing PARP degradation to promote apoptosis 37. However, resistance mechanisms also exist. For example, ABT-737 induces apoptosis by targeting anti-apoptotic Bcl-2 proteins, disrupting the mitochondrial membrane, and activating caspases, but it also activates TOPK, which in turn upregulates survivin and confers resistance to ABT-737 in cancer cells 93.

Autophagy, a process that maintains cellular homeostasis under stress conditions, also interacts closely with TOPK, and its deregulation contributes to cancer progression 94-96. Mechanistically, TOPK induces chemotherapy resistance through autophagy. It interacts with ULK1 and inhibits autophagy by phosphorylating ULK1 at Ser469, Ser495, and Ser533, thereby contributes to glioma resistance to temozolomide (TMZ). Inhibition of TOPK increases autophagic vacuoles, which are further enhanced by chloroquine, suggesting that TOPK regulates autophagy initiation 97. In cervical squamous cell carcinoma (CSCC), TOPK overexpression activates NF-κB and promotes autophagy, accelerating cancer progression, whereas knockdown of TOPK reduces NF-κB pathway activity and autophagy, thereby limiting migration and invasion 98. Likewise, in glioblastoma, TOPK regulates HDAC1 activity to activate NF-κB and contributing to the malignant phenotype 99. Additionally, TOPK blocks paclitaxel-induced autophagic cell death in H460 non-small cell lung cancer cells. Knockdown of TOPK increases apoptosis, autophagy, and p53 levels, enhancing paclitaxel-mediated apoptosis 100. Through the ERK/mTOR axis, TOPK further promotes autophagy and enhances cisplatin resistance. TOPK expression can also be transcriptionally regulated by EVI1, which directly targets the TOPK promoter 101.

### Role of TOPK in ferroptosis and anoikis

Ferroptosis, a regulated form of necrotic cell death, plays a role in cancer progression 102. Recent studies have shown that CLDN6 triggers Nrf2-mediated ferroptosis by regulating the TOPK-dependent AKT/GSK3 β/FYN axis and recruiting TOPK to the cell membrane for degradation by the UPS 103. Anoikis, a specialized form of programmed cell death caused by the loss of extracellular matrix attachment, represents another barrier to metastasis 104. Interestingly, TOPK has been linked to anoikis resistance in colorectal cancer (CRC). In this context, CXCL8 enhances resistance to apoptosis by activating AKT and ERK, with TOPK acting downstream of AKT. Thus, simultaneous inhibition of AKT, TOPK, and ERK may provide therapeutic strategies for CRC 90, 105.

### Role of TOPK in DNA damage repair

Oncogene activation disrupts DNA replication, thereby affecting replication fork progression and cell cycle timing 106. In line with this, TOPK inhibition impairs the DNA damage response, leading to increased DNA damage and decreasing cell survival 8. During the G1 phase, TOPK helps regulate phosphorylation events that are essential for maintaining genomic stability 14. Depletion of TOPK also results in replication fork stalling and collapse under stress. Furthermore, TOPK interacts with DNA damage response proteins such as CHK1 and Cdc25c, and its inhibition increases cancer cells' vulnerability to genotoxic stress, thereby enhancing sensitivity to radiation therapy 23, 107. Additionally, TOPK regulates p38α activity by phosphorylating MKP1, which stabilizes MKP1 and suppresses p38α signaling, ultimately supporting tumor growth under DNA damage conditions 108. Consistently, knockdown of TOPK impairs p38 activation and reduces cell motility in response to growth factors, further underscoring its role in DNA damage response and tumor progression 109, 110.

### Role of TOPK in cardiovascular and metabolic regulation

TOPK also regulates metabolic pathways during cancer cell progression. By interacting with signaling molecules, it reprograms cellular metabolism to support the energy and biosynthetic demands of rapidly proliferating cancer cells. For example, in mice and cells exposed to high glucose, Sevoflurane Postconditioning (SPostC) reduces myocardial injury, apoptosis, and oxidative stress, which is accompanied by increased phosphorylation of TOPK, PTEN, and AKT. Inhibition of TOPK or the PI3K/AKT pathway blocks these protective effects, indicating that SPostC protects the heart through TOPK-mediated PTEN/PI3K/AKT activation 111. In pancreatic β-cells, ectopic expression of TOPK increases ERK1/2 phosphorylation, thereby promoting insulin secretion. TOPK expression is also epigenetically regulated by JunD and HDAC3, and inhibition of JunD improves β-cell proliferation and glucose tolerance in diabetic mice 112, 113. During pregnancy, TOPK upregulation is linked to impaired glucose tolerance and reduced β-cell proliferation when its kinase activity is inhibited 114. Additionally, PBK/TOPK mediates geranylgeranylation signaling to promote breast cancer cell proliferation 115.

### Role of TOPK in inflammatory and the tumor microenvironment

TOPK mediates lipopolysaccharide (LPS)-induced migration and invasion of breast cancer cells by activating the TLR4 signaling pathway, which increases TOPK expression. Mechanistically, depletion of TOPK reduces LPS-induced phosphorylation of p38 MAPK and suppresses NF-κB and MMP9 promoter activity, thereby inhibiting cancer cell migration and invasion. Elevated levels of TOPK and TLR4 in high-grade breast cancer tissues further support their involvement in tumor progression, positioning TOPK as a promising therapeutic target for inflammation-driven cancer metastasis 78. Furthermore, TOPK regulates inducible nitric oxide synthase (iNOS) and nitric oxide production in response to LPS, acting as a key effector in the LPS/TLR4-mediated signaling cascade. It promotes IκBα phosphorylation and NF-κB activation, which are critical for iNOS transcription. Knockdown of TOPK or inhibition of the NF-κB binding site decreases transcriptional activity in response to LPS 116. In addition, the PI3K/AKT/PBK pathway is involved in neutrophil extracellular trap (NET) formation, as observed in co-cultures of neutrophils and OSCC cells. TOPK contributes to enhanced NETs formation through changes in the PI3K/AKT/PBK pathway protein expression 117, 118. Similarly, in psoriasis, TOPK regulates neutrophil chemokines such as CXCL1, CXCL2, and CXCL8 by activating STAT3 and NF-κB p65 in keratinocytes, thereby promoting neutrophil infiltration and psoriasis progression 119. Inhibition of TOPK alleviates psoriasis-like dermatitis by regulating neutrophils infiltration 120. The tumor microenvironment (TME) represents a major barrier to cancer therapy, with cancer-associated fibroblasts (CAFs) driving tumor progression, drug resistance, and immune suppression. In kidney renal clear cell carcinoma, TOPK overexpression is strongly associated with reduced cytotoxic immune cell infiltration, increased immune checkpoint expression, and enhanced infiltration of immunosuppressive cells such as MDSCs and CAFs, underscoring its prognostic and therapeutic potential 121, 122. Moreover, TOPK enhances microglia/macrophage M2 polarization by inhibiting HDAC1/HDAC2 activity, potentially contributing to its neuroprotective effects against cerebral ischemia-reperfusion injury 99. Taken together, these findings confirm the involvement of TOPK in inflammatory processes.

### The expression of TOPK regulated by transcription factors

PBK/TOPK plays a crucial role in determining cell fate, with its transcription regulated by several transcription factors and signaling pathways (Figure 4). For instance, E2F1, a key cell cycle transcription factor, collaborates with c-Myc to activate PBK/TOPK transcription, thereby promoting cell cycle progression 123-125. In addition, CREB/ATF binds to the PBK/TOPK promoter to upregulate its expression 126. During mitosis, CDK1/cyclin B1 inactivates PP1α, leading to TOPK activation through autophosphorylation, which highlights the role of CDK1/cyclin B1 in regulating TOPK during cell division 51. In leukemia, TOPK is regulated by PP2A and BCR/ABL, where PP2A associates with and dephosphorylates TOPK, while BCR/ABL upregulates its expression. Conversely, inhibition of BCR/ABL by imatinib or activation of PP2A reduces TOPK phosphorylation, suggesting that TOPK functions as a downstream target of BCR/ABL signaling 127. Up-frameshift protein 1 (UPF1) promotes PBK transcription, as PBK transcriptional activity decreases upon UPF1 knockdown and increases with UPF1 overexpression. Consequently, UPF1 regulates PBK mRNA and protein levels and modulates FOXO1 expression through the PBK signaling axis 128.

## Targeting TOPK for cancer therapy

The oncogenic roles of TOPK highlight its potential as a therapeutic target to inhibit cancer progression and improve patient outcomes. In this section, we review the effects of targeting TOPK and its role in chemotherapy resistance.

### Inhibition of TOPK could arrest cancer growth

Targeting TOPK or suppressing its expression can be a promising strategy for cancer treatment. For example, inhibitors such as atorvastatin or GGTase I inhibitors (GGTI-298) suppress proliferation of ER-negative breast cancer MDA-MB-231 cells by targeting PBK/TOPK, indicating PBK/TOPK as a downstream effector of geranylgeranyl signaling. Additionally, PBK/TOPK inhibition reduces MDA-MB-231 cell proliferation, while YAP, a key Hippo signaling effector, regulates PBK expression, linking TOPK to Hippo-YAP signaling 115. In prostate cancer, TOPK induces androgen receptor splice variant ARv7 expression, promoting androgen independence. Silencing TOPK sensitizes prostate cancer cells to androgen receptor-targeted therapies, demonstrating its role in ARv7 regulation 129. Similarly, in high-grade lymphomas, PBK/TOPK overexpression forms part of a c-Myc-E2F1-PBK axis, and targeting this pathway reduces lymphoma cell growth and survival 124. In medulloblastoma, LIN28B regulates TOPK and its inhibition reduces tumor viability and growth 130. In chronic myeloid leukemia (CML), TOPK is regulated by BCR/ABL, and its inhibition with OTS514 suppresses cell proliferation and colony formation, underscoring its therapeutic relevance 127. In Ewing sarcoma, inhibiting the EWS-FLI1 fusion oncogene reduces PBK/TOPK expression, suggesting it is a directly target gene 131. Moreover, PRPK, a cancer-related protein, is phosphorylated by TOPK. Knockdown of TOPK reduces PRPK phosphorylation, conferring resistance to SSL-induced skin cancer in mice. Inhibiting PRPK reduces skin hyperplasia, angiogenesis, and cutaneous squamous cell carcinoma by blocking PRPK activation and lowering expression of proliferation and tumor markers such as cyclin D1, COX-2, and MMP-9 132. At the molecular level, TOPK interacts with JNK-interacting protein 1 to enhance JNK1 activity, which is essential for AP-1 transcription and cell transformation induced by UVB or H-Ras 65. Interestingly, TOPK has been identified as a potential target, showing promise for inhibiting SARS-CoV-2 133.

### Targeted TOPK could affect the cancer immune response

TOPK also modulates immune responses in cancer. In CRC, TOPK expression negatively correlates with immunosuppressive cells like Tregs and M2 macrophages, but positively associating with cytotoxic T cell-related genes 134. In RCC, TOPK activates PD-L1 expression, promoting immune evasion and inhibiting CD8^+^ T cell infiltration. Its inhibition enhances CD8^+^ T cell infiltration and improves anti-PD-L1 treatment efficacy 135. In skin cancer, a dual-target system incorporating the TOPK inhibitor OTS964 and albendazole (ABZ) demonstrate synergistic cytotoxic effects 136. In addition, TOPK inhibition enhances IFN-γ and TNF-α production in NK-92MI cells, thereby improving antitumor activity 137. In ESCC, TOPK regulates the tumor microenvironment by promoting immune cell invasion and microsatellite instability 138. Similarly, TOPK, CCNA2, and KIF4A collectively regulate the tumor microenvironment in HCC, influencing immune cell invasion and microsatellite instability (MSI) 139. Furthermore, in CAR T-cell therapy, TOPK inhibition enhances T cell proliferation, memory formation, and immune checkpoint regulation 140. Immune infiltration analysis also showed that TOPK-induced immune escape may involve altered antigen presentation, dendritic cells, and CD8^+^ T cell infiltration 141. Mechanistically, TOPK phosphorylates MSL1, enhancing its interaction with MSL2, MSL3, and KAT8, which enriches the MSL complex at the CD276 promoter. This increases histone H4 K16 acetylation and activates CD276 transcription, a molecule regulated in NPC and correlates with immune infiltration, suggesting that the PBK/MSL1/CD276 signaling axis contributes to immune evasion 142.

### Targeted TOPK could enhance cancer cells sensitivity to chemotherapy

TOPK is also implicated in chemotherapy resistance. For instance, methylseleninic acid (MSeA) overcomes gefitinib resistance in NSCLC through the asparagine-MET-TOPK axis 143, while ERK2-mediated phosphorylation of TOPK enhances RCC cell sensitivity to sorafenib 52. In NSCLC, the COX2-TXA2 pathway activates MET and TOPK phosphorylation, promoting gefitinib resistance 144. Moreover, combining TOPK inhibitors with targeted therapies improves therapeutic sensitivity and delays drug resistance. TOPK interacts with c-Jun to influence the response of lung cancer to EGFR inhibitors gefitinib, with silencing TOPK enhancing drug sensitivity 145. The TOPK inhibitor pantoprazole, in combination with celecoxib and gefitinib, induced apoptosis in gefitinib-resistant lung cancer cells and suppressed tumor growth 144. In addition, TOPK enhances doxorubicin resistance by positively regulating NF-κB activity in TRAIL signaling, thereby promoting anti-apoptotic gene expression 146. MicroRNA-216b (miR-216b) downregulates TOPK in lung adenocarcinoma, increasing oxaliplatin sensitivity 147. In HCC, PBK mediates oxaliplatin resistance by regulating PTEN, while in ovarian cancer, PBK interacts with TRIM37 to promote PARPi resistance in ovarian cancer 148, 149. Notably, combining HI-TOPK-032 with alectinib, a first-line therapy for ALK-positive lung cancer, improves treatment sensitivity 150. Finally, TOPK ablation sensitizes cells to TRAIL-induced apoptosis 151, while, CHIP, an E3 ubiquitin ligase, reduces PBK stability through the ubiquitin-protease pathway, inhibiting ERK pathway and suppressing NSCLC radio-resistance 152, 153. In glioblastoma, PBK inhibition improves radiotherapy efficacy by regulating CCNB2, a key factor in tumorigenesis and radio-resistance 154. Collectively, these findings demonstrate that targeting TOPK not only suppresses tumor growth but also modulates immune responses and reverses drug resistance. Thus, TOPK inhibition (either as monotherapy or in combination with existing targeted drugs) holds significant promise for clinical translation and may ultimately improve cancer patient outcomes.

### Inhibitors of TOPK in cancer treatment

**Figure 5:**
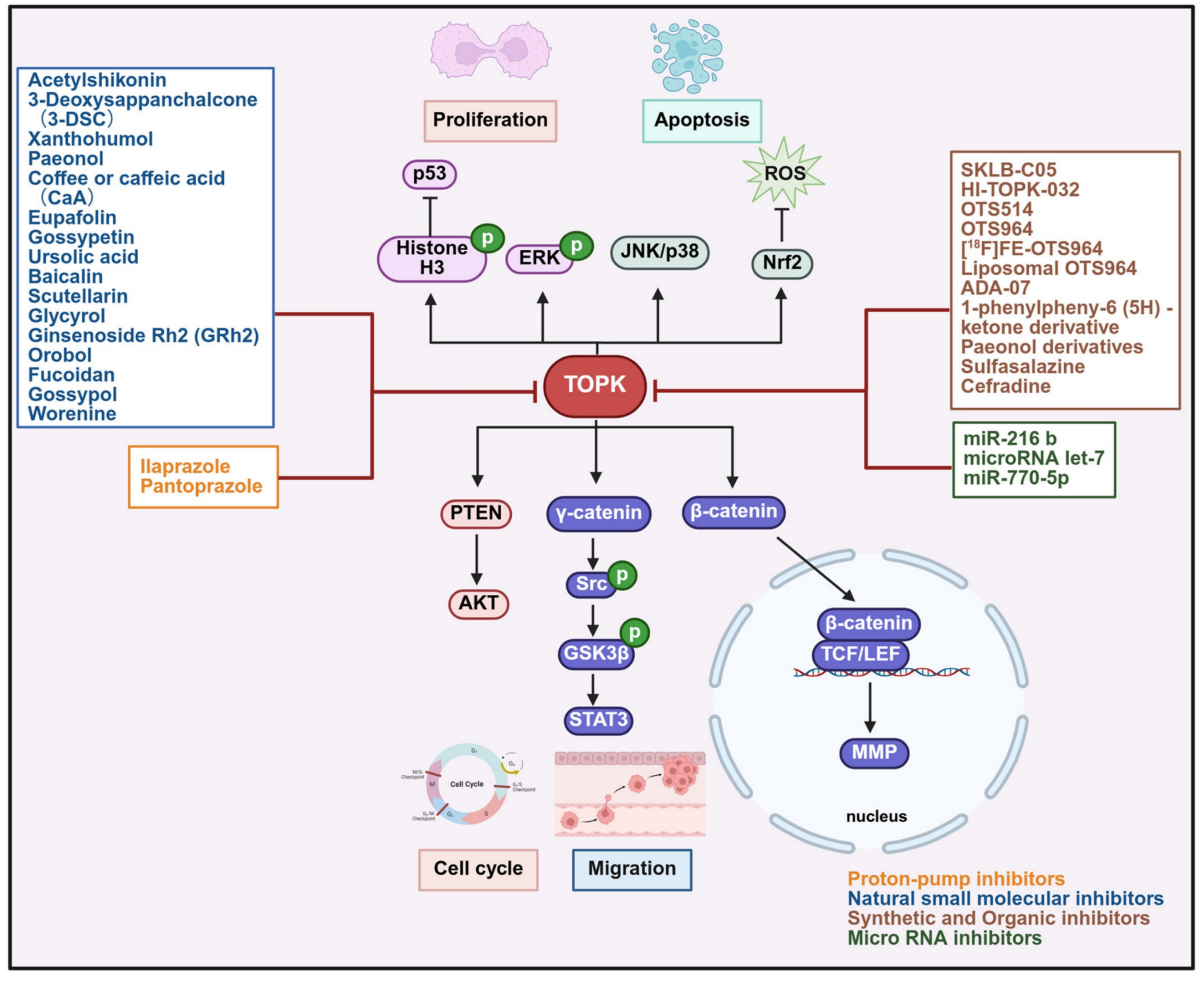
Schematic representation of TOPK inhibitors and their mechanisms of action in cancer.

Targeted therapy is a promising approach in drug design, and the upregulation of TOPK has been linked to cancer diagnosis and prognosis, making it an important therapeutic target. To date, several effective TOPK inhibitors have been developed and can be categorized into four main groups: synthetic and organic compounds, proton pump inhibitors, natural small molecular inhibitors and microRNAs (Figure 5, Table 2-5). Notable inhibitors include HI-TOPK-032 10, OTS514 155 and OTS964 156, alongside organic compounds like sulfasalazine 157 and proton pump inhibitors like pantoprazole 158 and ilaprazole 159, some of which are already FDA-approved drugs. In this section, we provide a comprehensive summary of the mechanisms of action of various TOPK inhibitors, offering valuable insights and potential strategies for their future clinical application in cancer therapy.

### Synthetic and biologically derived inhibitors of TOPK

Given its critical role in tumorigenesis, metastasis, and poor prognosis, targeting TOPK has emerged as a viable therapeutic strategy. HI-TOPK-032, OTS514, OTS964, and SKLB-C05 are representative synthetic inhibitors of TOPK (Table 2). For instance, HI-TOPK-032, developed by our group, specifically targets TOPK and suppresses diverse tumor types by inhibiting ERK and RSK phosphorylation through AP-1 or p53 pathways. In mouse xenograft models, treatment with HI-TOPK-032 (1-10 mg/kg) inhibited tumor growth by more than 60% without evident toxicity 10, 140. Similarly, the TOPK inhibitor OTS514 suppresses cancer cell proliferation by downregulating E2F target genes and inhibiting FOXM1 and MELK activities, thereby inducing cell cycle arrest and apoptosis through the p53 signaling pathway. It exhibits potent growth-inhibitory effects in multiple tumor types, including oral squamous carcinoma and small-cell lung cancer 155, 160. OTS964, another TOPK inhibitor, causes cell division defects and apoptosis 74, 156. Additionally, its radiolabeled derivative [^18^F] FE-OTS964 has been developed as an imaging agent 161, while liposomal OTS964 shown enhanced fluorescence when bound to albumin 162. ADA-07 binds TOPK to inhibit ERK1/2, p38, JNK phosphorylation, and AP-1 activity 163. SKLB-C05 inhibits colorectal carcinoma growth and metastasis by downregulating TOPK-mediated signaling, and blocks the FAK/Src-MMPs pathway 164. Moreover, structural optimization has led to phenanthridinone derivatives with stronger anti-TOPK activity than OTS964 165. OTS964, itself has been reported to suppress tumor growth in ovarian and lung cancers 49, 156, and to enhance radiosensitivity 166, 167. However, acquired resistance to OTS964 remains a major challenge. Other compounds, such as paeonol derivatives, inhibit TOPK-related signaling by modulating MAPK and NF-κB pathways, as well as DNA damage-related proteins such as H2AX and STAT3 168. Among repurposed drugs, sulfasalazine inhibits TOPK, reduces p-AKT, and suppresses tumor proliferation and metastasis through the PI3K/AKT pathway, with significant *in vivo* antitumor effects at concentrations up to 150 μM 157. Cefradine, another agent, directly binds TOPK and inhibits T-LAK cell-derived protein kinase. Importantly, cefradine showed no cytotoxicity in HaCat or JB6 cells, while treatment at 100 mg/kg markedly reduced epidermal thickness and attenuated immune cell infiltration in mice 169.

### Targeting TOPK with proton pump inhibitors

Proton pump inhibitors (PPI) are widely prescribed drugs 170, 171. Interestingly, pantoprazole and ilaprazole also function as TOPK inhibitors (Table 3). In preclinical models, pantoprazole at 100 mg/kg significantly suppressed xenograft tumor growth, while oral administration of ilrazole at 75 and 150 mg/kg inhibited tumor growth without causing hepatotoxicity, nephrotoxicity, or weight loss 158, 159. In addition, pantoprazole sensitizes gefitinib-resistant NSCLC cells to apoptosis by directly targeting TOPK 144.

### Small-molecule inhibitors of TOPK from natural sources

Natural compounds, owing to their diverse chemical structures, can modulate multiple cellular pathways by inhibiting proliferation, inducing apoptosis, or regulating inflammation. Importantly, they also enhance drug sensitivity, suppress tumor growth and metastasis, and show synergistic effects with conventional therapies 172-175. Among them, acetylshikonin, a bioactive compound from *Lithospermum erythrorhizon* root, directly binds the ATP pocket of TOPK, inducing G1 arrest, inhibiting colon cancer cell proliferation, and promoting apoptosis. Mechanistically, it blocks TOPK-mediated signaling by reducing the phosphorylation of ERK, RSK, c-Jun, as well as NF-κB activity. *In vivo*, acetylshikonin (120 or 160 mg/kg) significantly reduced tumor growth without affecting body weight 1. Likewise, 3-Deoxysappanchalcone (3-DSC) induces G2/M cell cycle arrest and apoptosis in colon and skin cancers by targeting TOPK 2, 176. Xanthohumol, a principal prenylated chalcone from hops, also directly targets TOPK and inhibits its activity, thereby reducing phosphorylation of histone H3 and AKT *in vitro* and *in vivo*, which contributes to its anticancer effects 177. Other natural agents also exhibit inhibitory effects on TOPK. For example, paeonol reduces SUV-induced inflammation by targeting TOPK 178. Caffeic acid and coffee inhibit colon cancer metastasis by suppressed MEK1/TOPK and ERK/AP-1 signaling 179. Eupafolin decreased histone H3 and Ki67 expression while enhancing caspase 3 activity in tumor tissues 180. Gossypetin inhibits PBK/TOPK activity and downregulates the phosphorylation of PBK/TOPK and p38 MAPK 181. Ursolic acid (UA), a triterpene found in apples, rosemary, and basil, inhibits TOPK phosphorylation in a concentration-dependent manner, activating the p53-p21 pathway and inducing G1 cell cycle arrest in breast cancer and colorectal cancer 182. Additionally, gossopol promotes differentiation through the PBK/TOPK pathway 183. Other flavonoids, such as baicalin, scutellarin, and glycyrol, have also been reported to inhibit TOPK activity 184-190. Beyond oncology, natural inhibitors may also regulate inflammation conditions through TOPK inhibition. For example, worenine, a compound from the rhizome of *Coptis chinensis*, inhibits TOPK activity and alleviates M5- and IMQ-induced psoriasis-like dermatitis by reducing neutrophil infiltration 120.

### Targeting TOPK with microRNA inhibitors

MiRNAs regulate diverse processes in cancer, including proliferation, apoptosis, invasion, and angiogenesis 191. Notably, miR-216b downregulates TOPK by binding to its 3' untranslated region (3' UTR) 147, whereas miR-216b-3p suppresses lung adenocarcinoma cell proliferation by targeting TOPK, leading to increased p53 and p21 expression and inhibition p38 MAPK activation 192. Additionally, let-7 inhibits PBK to reduce cell proliferation 130, and miR-770-5p sensitizes cells to radiation by targeting TOPK and increasing apoptosis (Table 5) 193.

## Conclusions

In summary, as one of the kinases in the MAPKK family, TOPK plays a crucial role in cell cycle regulation and mitotic progression, influencing a wide range of cellular functions and activating key signaling pathways, such as MAPK and PI3K/AKT. Its overexpression is strongly associated with tumor growth, metastasis, drug resistance, and it is notably upregulated in most cancer tissues. Collectively, these findings suggest that TOPK drives tumorigenesis and development by regulation cell proliferation, kinase-mediated signaling, immune modulation, and metabolic pathways. However, its biological role appears to be context-dependent, varying across cancer types and potentially shaped by tissue-specific or mutational factors. Although database analyses indicate that TOPK is highly expressed in multiple cancers, including ACC, BLCA, CESC, LGG and TYHM, experimental validation remains limited, and the precise oncogenic mechanisms in these malignancies are still unclear. Moreover, the role of TOPK in metabolic regulation and post-translational modifications has scarcely been investigated. Future studies should therefore aim to bridge these gaps by employing advanced approaches such as single-cell transcriptomics, spatial transcriptomics, metabolomics, and proteomics of post-translational modifications. From a therapeutic perspective, targeting TOPK presents a promising strategy in cancer treatment. As targeted therapies continue to advance, inhibiting kinase phosphorylation to disrupt aberrant signaling cascades remains a central focus of anticancer drug development. Nevertheless, despite extensive preclinical evidence demonstrating the important of TOPK in tumor growth and progression, and the availability of multiple inhibitors capable of suppressing its kinase activity, there are currently no specific TOPK-targeted drugs approved for clinical use. Therefore, further investigation is required to evaluate their therapeutic efficacy in patients, and combining strategies with other clinal drugs may offer synergistic benefits. In addition, the role of TOPK in chemotherapy resistance warrants deeper exploration to better understand its contribution to treatment failure and to identify novel approaches for overcoming resistance. Overall, more focused and integrative research is essential to fully elucidate the biological functions of TOPK and to realize its potential as a viable therapeutic target in cancer therapy.

## Supplementary Material

Supplementary figures.

## Figures and Tables

**Table 1:** The role of TOPK in different types of cancer

	Cancer Type	Samples (Number of cases)	Quantity of expression	Experiments	Statistical influencing factors (P values)	References
	Bladder cancer	Tissues (76)	Upregulation	QRT-PCR, IHC	Stage (*), grade (*), type of bladder cancer (*)	32
	Breast cancer	Tissues (202)	Upregulation, with poor prognosis	IHC	Histological (*), TNM (*), lymph node metastasis (*), number of lymph node (*), estrogen receptor (ER *), progesterone receptor (PR *), human epidermal growth factor receptor 2 (HER-2 *), Ki-67 (*), molecular typing (*)	33
		Triple-negative breast cancer (TNBC) patient tissues before and after of neoadjuvant chemotherapy (NACT) with docetaxel + epirubicin + cyclophosphamide (TEC) drugs (66)	Upregulation, with poor prognosis and poor response to treatment	IHC	The Miller-Payne (MP) system (*)	34
	Cervical cancer	Tissues (170)	Upregulation	IHC	Age (*), histological (*), differentiation (*), lymph node metastasis (*), vaginal and cervical invasion (*), TNM (*), tumor size (*), and type of cervical cancer	35
	Cholangiocarcinoma	Tissues (74)	Upregulation, with the low expression with poor of PBK/TOPK is predicative of poor survival	IHC	Differentiation (*), gender (*)	36
	Chordoma	Tissues (55)	Upregulation, with poor prognosis	IHC	Recurrence and metastasis (*), disease status (*)	37
	Colorectal cancer	Tissues (1420, 162 and 269)	Upregulation in both cytoplasm and nucleus	IHC, fluorescent immunohistochemistry	Age (*), differentiation (*), T value (*), tumor location (*), grade (*), stage (*), histological subtype (*), advanced disease stage (*), metastasis (*), low cytoplasm PBK/TOPK expression, negative nuclear PBK/TOPK expression, and low total PBK/TOPK expression were significantly associated with poor overall patient survival, Ki-67 (*), mutation of KRAS/BRAF	38-40
	ESCC	Tissue (54), ESCC cell lines (15)	Upregulation with poor survival	IHC,	Macroscopic appearance (*), TNM classification of T category (*), macroscopic (*), tumor depth (*), tumor size (*), venous and lymphatic invasion (*)	41
	Hepatocellular carcinoma	Tissues (33 cases of hepatocellular carcinoma and 10 cases of normal liver tissue)	No expression	IHC	-	36
	Gastric cancer	Tissues (144) and GC cell lines (5)	Upregulation	IHC, QRT-PCR	Size (*), stage (*), venous invasion (*), TNM classification of pT category (*) and pN category (*), recurrence (*), invasion depth (*), p53 (DO7)	42
		Tissues (385)	Upregulation with poor survival	IHC, QRT-PCR	Size (*), location, gross type, histological type, stage (*), invasion depth (*), perineural invasion, lymphovascular emboli, lymph node metastasis (*)	43
Lymphoma		Tissues (20)	Upregulation of p-TOPK with short PFS	IHC	Karnofsky performance status (KPS) (*), ocular involvement, deep brain structure involvement, number of lesions (*), chemotherapy and radiotherapy, etc	44
		Tissues (2), Burkitt lymphoma cell lines (8), other tumor cell lines (10)	Upregulation	PCR, Northern analysis	-	45
Lung adenocarcinoma		Tissues (203)	Upregulation with poor survival	IHC	Age (*), smoking history, tumor size (*), differentiation, necrosis (*), angiolymphatic invasion (*), TOPK IHC score (>3) (*)	46
	Oral cancer	Tissues (287)	Upregulation, mainly localized in the cytoplasm	IHC	Smoking, betel nut, alcohol consumption, differentiation, stage (*)	47
	Osteosarcoma	Tissues (66)	Upregulation with poor survival	IHC	Tumor location, histological grade, recurrence, metastasis (*), disease status (*)	48
	Ovarian cancer	Tissues (163 of EOC and 26 of borderline tumor)	Upregulation with poor progression-free survival and overall survival	IHC	Grade (*), lymph node metastasis, Ki-67 (*) and cytological examination	49
	Testicular Cancer	GEO datasets (GSE3218 and GSE1818)	Downregulation	-	-	28
						

**Table 2:** Synthetic and organic inhibitors of TOPK

Inhibitors	Structure	Mechanism of action	Types of cancer	Classification	References
HI-TOPK-032	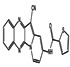	Inhibition of phosphorylation of ERK and ERK direct downstream proteins ERK and RSK, or through AP-1 or p53 signaling pathways	Colorectal cancer		10
OTS514	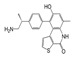	FOXM1 and MELK activities were inhibited, and cell cycle arrest and apoptosis were induced	Small cell lung cancer		155
		OTS514 inhibited the proliferation of OSCC cells by down-regulating the expression of E2F target genes, and induced cell apoptosis by mediating the p53 signaling pathway	Oral squamous carcinoma		160
OTS964	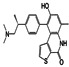	Inhibition of TOPK activity inhibits tumor growth by inducing defects in cell division and inducing apoptosis	Lung cancer		156
[^18^F] FE-OTS964	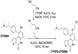	The first TOPK inhibitor for imaging purposes	Glioblastoma		161
Liposomal OTS964	-	The simple method of association of OTS964 with liposomes, when bound to albumin, relies on enhanced OTS964 fluorescence			162
SKLB-C05	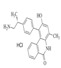	SKLB-C05 accelerated apoptosis by targeting members of the Bcl-2 family, including Mcl-1, Bcl-2 and Bax, with the expression of both cyclin B1 and CDK1 inhibited	Colorectal cancer	Synthetic compounds	164
ADA-07	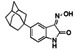	ADA-07 directly binds to TOPK and inhibits TOPK kinase activity, and inhibits ERK1/2, p38 and JNKs phosphorylation, thereby inhibiting AP-1 activity	Skin cancer		163
1-phenylpheny-6 (5H) -ketone derivative	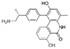	The growth of cancer cells was inhibited by apoptosis and the activity of TOPK was specifically inhibited.	Colorectal cancer		165
Paeonol derivatives	_	Paeonol derivatives interacts with TOPK, regulates its downstream pathways MAPK and NF-κB, and inhibits the expression of DNA damage-related protein H2AX and proliferation-related protein STAT3	Psoriasiform skin inflammation		168
Sulfasalazine	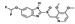	Sulfasalazine directly binds TOPK to reduce the level of p-AKT, and affects the PI3K/AKT signaling pathway, inhibit the proliferation and metastasis	Thyroid cancer	Organic compounds (FDA approved)	157
Cefradine	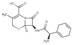	Cefradine can directly bind to TOPK, block solar UV-induced skin inflammation by directly inhibiting T-LAK cell-derived protein kinase	Skin inflammation and skin cancer	Organic compounds (FDA approved)	169

**Table 3:** Proton pump inhibitors of TOPK

Inhibitors	Structure	Mechanism of action	Types of cancer		References	
Pantoprazole	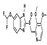	Direct binding to TOPK to inhibit TOPK activity* in vitro* and *in vivo* strongly reduced the phosphorylation of histone H3 (Ser10)	Colorectal cancer	FDA-approved	158	
Ilaprazole	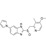	Direct binding of TOPK to inhibit TOPK activity resulted in strongly reduced phosphorylation of histone H3 (Ser10), a downstream substrate of TOPK, in cancer cells	Lung cancer, colon cancer, human ovarian cancer, and pancreatic cancer cells	FDA-approved	159	

**Table 4:** Natural small molecular inhibitors of TOPK

Inhibitors	Structure	Mechanism of action	Types of cancer	Source	References
Acetylshikonin	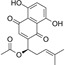	Direct inhibition of TOPK activity, interacting with the ATP-binding pocket of TOPK, inhibits cell proliferation by inducing cell cycle arrest in G1 phase, stimulates apoptosis, and increases the expression of apoptotic biomarkers in colorectal cancer cell lines	Colorectal cancer	The main bioactive compounds present in the roots of *Lithospermum erythrorhizon*	1
3-Deoxysappanchalcone (3-DSC)	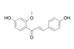	3-DSC inhibits colon cancer cell growth and induces G2/M cell cycle arrest and apoptosis by directly targeting the TOPK-mediated signaling pathway	Colorectal cancer, Skin Cancer	An ingredient of *Caesalpinia sappan* L. is a natural oriental medicine	2, 176
Xanthohumol	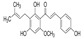	Direct interaction with TOPK reduced the phosphorylation of TOPK kinase activity and its downstream signals histone H3 and Akt	Non-small cell lung cancer	The major isoprenylated chalcones isolated from hops	177
Paeonol	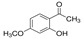	Paeonol has a protective effect on SUV-induced inflammation by targeting TOPK	Inflammation of the skin	Isolated from traditional Chinese herbs, such as danpi, paeoniflora root, and Japanese wild peony	178
Coffee or caffeic acid (CaA)	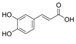	Targeting MEK1 and TOPK inhibited colon cancer metastasis and tumor cell transformation, inhibited ERKs phosphorylation, AP-1 and NF-κB transactivation, and subsequently inhibited TPA, EGF and H-ras-induced tumor transformation in JB6 P+ cells	Colorectal cancer		179
Eupafolin	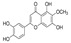	Eupafolin treatment decreased histone H3 and Ki-67 and increased caspase-3 in tumor tissues	Esophageal cancer	The main active ingredient extracted from the traditional Chinese medicine *Artemisia vulgaris* L.	180
Gossypetin	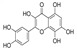	Gossypetin inhibited the activity of PBK/TOPK and inhibited the phosphorylation of PBK/TOPK, p38 MAPK, ERK1/2 and H2AX induced by solar UV	Basal cell carcinoma of the skin	Flavonoids derived from hibiscus, a traditional Chinese medicine	181
Ursolic acid	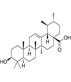	Ursolic acid could inhibit mouse minute-2 protein (MDM2) and T-LAK cell-derived protein kinase (TOPK), two negative regulators of p53, leading to ursolic acid-induced p53 activation	Breast cancer, colon cancer	apple, rosemary, and holy basil	182
Gossypol	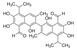	Gossypol could induce differentiation in leukemic cell lines by specifically inhibiting the phosphorylation of PBK/TOPK without affecting total protein levels	Leukemia	Cottonseed (gossypium) plants	183
Baicalin	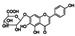	Direct binding to PBK/TOPK inhibits TOPK activity, and PBK/TOPK downstream signaling molecules histone H3 and ERK2 are also reduced	Lung cancer	The main bioactive component extracted from the root of Baical Skullcap	184
Scutellarin	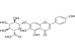	Direct binding to TOPK* in vitro*, inhibit the activity of TOPK, and inhibit the phosphorylation of extracellular regulated protein kinase 1/2 (ERK1/2) and histone H3 in cells	Melanoma	The active ingredient extracted from *Erigeron breviscapus* (Vant.) Hand-Mazz.	185
Glycyrol	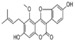	Glycyrol binds strongly to TOPK protein and inhibits its kinase activity, leading to activation of the apoptotic signaling pathway	Lung cancer	Representative coumarin compounds isolated from licorice	186
		Inhibition of iron cell apoptosis alleviates acute kidney injury	Acute kidney injury		190
Ginsenoside Rh2 (GRh2)	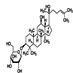	GRh2 could directly bind to PBK/TOPK, inhibit the activity of PBK/TOPK, inhibit the phosphorylation of extracellular regulated protein kinase 1/2 (ERK1/2) and (H3) in colorectal cancer cells, and inhibit the growth of xenograft tumors	Colorectal cancer	The main bioactive ingredient in American ginseng, a commonly used herbal medicine	187
Orobol	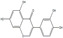	Direct binding to TOPK and inhibits TOPK kinase activity in an ATP-independent manner	Squamous cell carcinoma of the skin	Derived from soybean, it is present in trace amounts in natural and fermented foods	188
Fucoidan	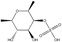	Fucoidan directly targeting TOPK and suppressing the TOPK/ERK1/2/MSK1 signaling axis, fucoidan effectively prevented EGF-induced tumor cell transformation in both* ex vivo* and *in vitro* models	Colon cancer	*Fucus evanescens*	189
Worenine	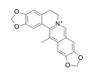	Worenine inhibits TOPK activity to alleviate psoriasis-like dermatitis induced by M5	Psoriasiform dermatitis	*Coptis chinensis*	120

**Table 5:** MicroRNA inhibitors of TOPK

Inhibitors	Mechanism of action	Types of cancer	References
miR-216 b	MiR-216b was negatively correlated with the expression of TOPK, and it could down-regulate the level of TOPK by binding to the 3' untranslated region (3' UTR) of TOPK	Colorectal cancer	147
miR-216b-3p	Overexpression of miR-216b-3p may increase the expression of p53 and p21 and prevent p38 MAPK activation	Adenocarcinoma of the lung	192
microRNA let-7	MicroRNA let-7 inhibits PBK to reduce cell proliferation	Medulloblastoma	130
miR-770-5p	Overexpression of miR-770-5p increases apoptosis by directly targeting PDZ-binding kinase (PBK) and ultimately sensitizing the radiation response *in vitro* and *in vivo*	MCF7, A549 and HCT-116 cells	193
