# Supplementary material for: The Roles of TOPK in Tumorigenesis and Development: Structure, Mechanisms, Pathways, and Therapeutic Implications
Source: Int J Biol Sci. 2026 Jan 1;22(1):327–45. doi: 10.7150/ijbs.122960 (PMC12681809; doi:10.7150/ijbs.122960)
Supplement: Supplementary file 1 — Supplementary figures. [file ijbsv22p0327s1.pdf]

Supplement Figure 1

A

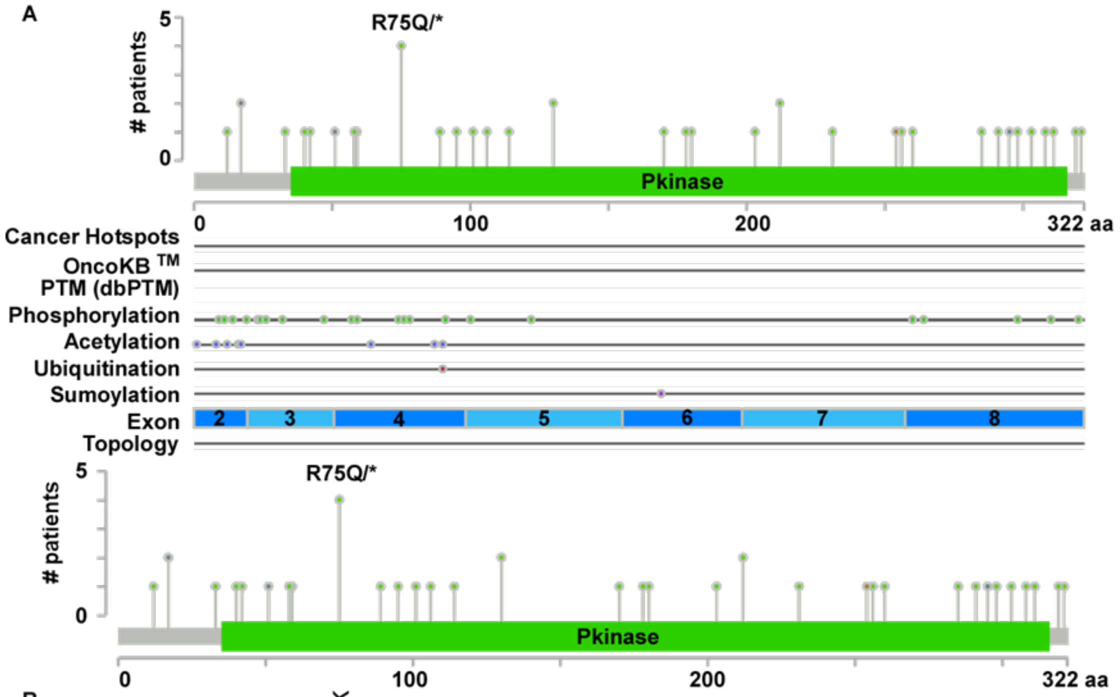

B

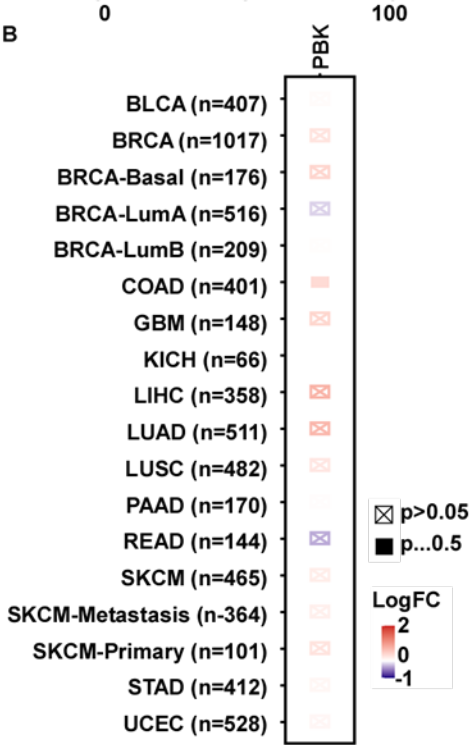

Supplement Figure 2

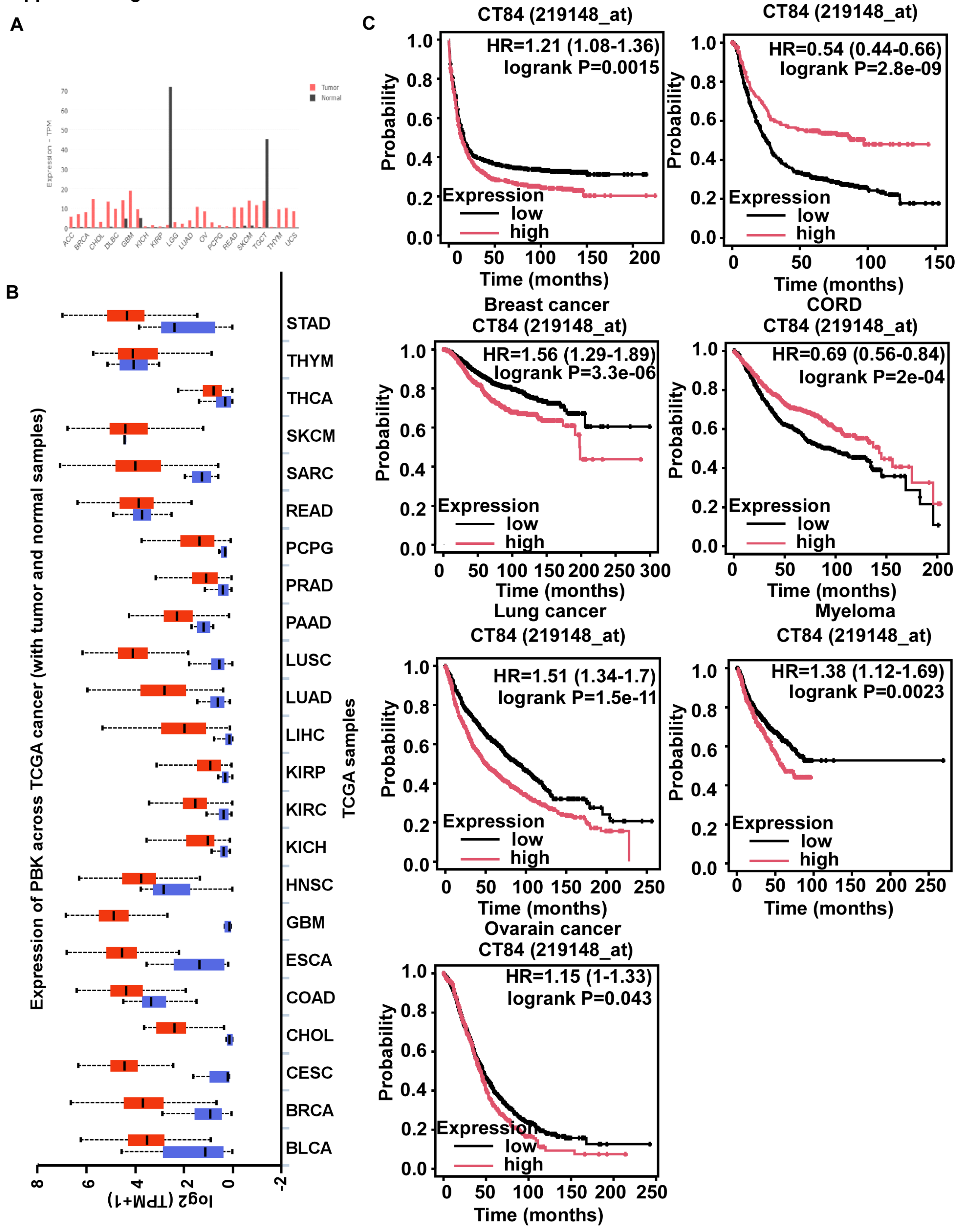

Supplement Figure 1. Genetic alterations of TOPK across cancer types based on analyses from the cBioPortal (https://www.cbioportal.org/) (A) and TIMER 2.0 databases (https://compbio.cn/timer2/) (B).

Supplement Figure 2. Expression levels of TOPK in various cancer types obtained from the GEPIA 2 (http://gepia2021.cancer-pku.cn/) (A) and UALCAN databases (https://ualcan.path.uab.edu/index.html) (B), and overall survival analysis of TOPK expression in different cancers using the Kaplan-Meier Plotter database (https://kmplot.com/) (C).
